# Survival Risk Prediction of Esophageal Squamous Cell Carcinoma Based on BES-LSSVM

**DOI:** 10.1155/2022/3895590

**Published:** 2022-07-06

**Authors:** Yanfeng Wang, Wenhao Zhang, Junwei Sun, Lidong Wang, Xin Song, Xueke Zhao

**Affiliations:** ^1^School of Electrical and Information Engineering, Zhengzhou University of Light Industry, Zhengzhou 450000, China; ^2^State Key Laboratory of Esophageal Cancer Prevention & Treatment and Henan Key Laboratory for Esophageal Cancer Research of the First Affiliated Hospital, Zhengzhou University, Zhengzhou 450066, China

## Abstract

Esophageal squamous cell carcinoma (ESCC) is one of the highest incidence and mortality cancers in the world. An effective survival prediction model can improve the quality of patients' survival. In this study, ten indicators related to the survival of patients with ESCC are founded using genetic algorithm feature selection. The prognostic index (PI) for ESCC is established using the binary logistic regression. PI is divided into four stages, and each stage can reasonably reflect the survival status of different patients. By plotting the ROC curve, the critical threshold of patients' age could be found, and patients are divided into the high-age groups and the low-age groups. PI and ten survival-related indicators are used as independent variables, based on the bald eagle search (BES) and least-squares support vector machine (LSSVM), and a survival prediction model for patients with ESCC is established. The results show that five-year survival rates of patients are well predicted by the bald eagle search-least-squares support vector machine (BES-LSSVM). BES-LSSVM has higher prediction accuracy than the existing particle swarm optimization-least-squares support vector machine (PSO-LSSVM), grasshopper optimization algorithm-least-squares support vector machine (GOA-LSSVM), differential evolution-least-squares support vector machine (DE-LSSVM), sparrow search algorithm-least-squares support vector machine (SSA-LSSVM), bald eagle search-back propagation neural network (BES-BPNN), and bald eagle search-extreme learning machine (BES-ELM).

## 1. Introduction

Cancer is one of the leading causes of human death in both developed and developing countries [1]. Esophageal cancer is the sixth leading cancer in the world, including esophageal squamous carcinoma and esophageal adenocarcinoma [2]. More than 90% of esophageal cancers are esophageal squamous cell carcinoma, and most of them are diagnosed in advanced stages [3]. The pathology of esophageal squamous cell carcinoma is complicated, and effective diagnosis and treatment strategies are lacking [4, 5]. In recent years, the incidence of esophageal squamous cell carcinoma has been on the rise, and the mortality rate remains high [6].

At present, with the continuous deepening of human research, the treatment methods and treatment concepts of ESCC have been continuously improved [7–9]. However, there is still a lack of marker models and prognostic index that can accurately and effectively reflect the prognosis of ESCC patients [10]. Generally, TNM staging is considered to be the best prognostic indicator for ESCC. However, patients with the same TNM stage often have different prognoses [11]. The TNM staging alone cannot accurately determine the patient's risk of death [12]. Therefore, it is important to establish a reasonable prognostic index.

In recent years, with the continuous progress of machine learning technology, more and more intelligent algorithms are proposed and applied in multiple fields [13–19]. A hybrid model of genetic algorithm (GA) and least-squares support vector machine (LSSVM) is used by Ahmadi and Chen [20] to predict the relevant experimental permeability reduction ratio due to scale deposition during water injection, and the results confirm the validity of the GA-LSSVM model. LSSVM is used by Ahmadi and Pournik [21] to build a predictive model for determining the chemical flooding efficiency of the oil reservoir, and the results show that the model has good stability and reliability. In [22], a method based on local mean decomposition and improved FA-optimized combined kernel least-squares support vector machine is proposed to predict short-term wind speed. The results show that the proposed LMD-FA-LSSVM model has better prediction performance.

In the medical field, the doctors' diagnosis is effectively aided by the application of many new algorithms. A combined classification and regression approach is proposed by Zhu et al. [23] for early diagnosis of COVID-19 and prediction of time to conversion in patients with severe symptoms. The results show that the accuracy of the proposed method in predicting severe cases reached 76.97% with a correlation coefficient of 0.524. In [24], a method combining extreme learning machine and gain ratio feature selection method is proposed and tested on the Wisconsin Breast Cancer Diagnostic (WBCD) dataset. The experimental results show that the accuracy of the proposed method reaches 0.9868. The genetic algorithm is used by Majid et al. [25] to select the best features and then use an ensemble classifier to predict gastric infections. The results show that the proposed method performs better than existing methods. In addition, random forest [26], extreme learning machines [27], BP neural networks [28, 29], and Elman neural networks [30] have achieved satisfactory results in the prognosis and diagnosis of certain cancers.

Compared with the above studies [24, 25, 27, 28] that mostly use genetic information and image information to predict patient mortality, the proposed work mainly has the following advantages. First, the patients' blood indicators and TNM staging indicators are used to predict the patient's survival status. Second, an effective prognostic index is established, which significantly improved the performance of the prediction model. Third, these machine learning algorithms rarely distinguish between patients of different ages. Due to differences in patient age, it is difficult for a single model to accurately predict the survival risk of all patients. Therefore, the goal of this article was to find a new set of indicators related to the survival of ESCC patients based on the patient's blood indicators and TNM staging information, establish reasonable prognostic indicators, and combine new machine learning techniques to predict the survival rate in patients of different ages.

In this study, seventeen blood indicators, age, and TNM staging information of 360 patients with ESCC are studied. Ten indicators related to patient survival are found through the feature selection method of genetic algorithm. The combination of these ten indicators has a significant correlation with the patient's survival, which is verified by the Cox regression method in the SPSS software. Using the binary logistic regression method, the prognostic index (PI) of patients with ESCC is constructed. The prognostic index (PI) is divided into four stages, and the different survival conditions of patients can be reasonably reflected in each stage. Comparing the PI staging system with the traditional TNM staging system, the results show that the PI staging system has a better AUC value. The ROC curve method is used to determine the critical threshold of patient age, and the patients are divided into the high-age groups and the low-age groups. Then, based on the Kaplan–Meier survival analysis, it is concluded that the low-age group has a better survival rate than the high-age group, which effectively reflects the survival status of different patients. Finally, the bald eagle search algorithm-least-squares support vector machine (BES-LSSVM) survival prediction model is further proposed in this study. The bald eagle search algorithm is used to optimize the parameters of the least-squares support vector machine, which improves the prediction accuracy of the model. The prognostic index (PI) and the above ten related indicators are used as inputs, and the five-year survival rate of the patient is used as output. The prediction accuracy rate of BES-LSSVM is better than the existing PSO-LSSVM, GOA-LSSVM, DE-LSSVM, SSA-LSSVM, BES-BP, and BES-ELM. Therefore, the method for survival prediction of patients with ESCC proposed in this study can accurately predict the survival level of patients.

The purpose of this article was to propose prognostic indicators PI and survival prediction models based on blood indicators and TNM staging information of patients with ESCC. Based on genetic algorithm feature selection, binary logistic regression, ROC curve, Kaplan–Meier survival analysis, Cox regression analysis, and BES-LSSVM, a method for predicting the survival risk of patients with ESCC is proposed. The main contributions of this article can be summarized as follows:

1. A combination of ten indicators is found based on genetic algorithm feature selection, which is verified to be significantly associated with survival in patients with ESCC.
2. The prognostic index of patients with ESCC is constructed by the binary logistic regression method, which can reasonably reflect the survival of patients at different stages.
3. The survival risk levels of patients with ESCC of different ages are gotten based on the ROC method, which can reasonably reflect the survival status of patients of different ages.
4. The BES-LSSVM is proposed and accurately predicts the five-year survival rate of patients with ESCC.

This work is presented as follows. In Section 2, the original data are analyzed, a combination of multiple indicators that is significantly related to patient survival is found, and prognostic index is constructed. The survival risk of patients of different ages is obtained. In Section 3, the bald eagle search-least-squares support vector machine is proposed, and the five-year survival rate of patients with ESCC is effectively predicted. In Section 4, the conclusions of this article are presented.

## 2. Feature Selection and Construction of Prognostic Indicators

### 2.1. Data Introduction

The clinical data of 360 patients with ESCC used in this article are from patients who were treated in the First Affiliated Hospital of Zhengzhou University from January 2007 to December 2018. The clinical information includes seventeen blood indicators, age, and TNM staging information. The seventeen blood indicators are as follows: white blood cell count (WBC), lymphocyte count (LYMPH), globulin (GLOB), prothrombin time (PT), albumin (ALB), red blood cell count (RBC), thrombin time (TT), basophil count (BASO), eosinophil count (EO), international normalized ratio (INR), neutrophil count (NEUT), total protein (TP), monocyte count (MONO), fibrinogen (FIB), hemoglobin concentration (HGB), platelet count (PLT), and activated partial thromboplastin time (APTT). Among all patients, 177 patients survived more than five years and 183 patients survived less than five years, and the data are evenly distributed. The end points are the time of death after treatment and the end of follow-up. The population proportion information of the dataset is shown in Table 1. Information on seventeen blood indicators is shown in Table 2.

### 2.2. Feature Selection Based on Genetic Algorithm

A genetic algorithm (GA) is a global optimization adaptive probability search algorithm [31]. GA has the characteristics of group search, which makes it easy to jump out of the local optimum [32]. Therefore, it is often selected as the search algorithm with better feature selection. In many studies, GA is used as a wrapper feature selection technique [33]. In this study, 17 blood indicators and TNM staging information of patients with ESCC are used as independent variable, and the five-year survival rate of patients is used as dependent variable. The least-squares support vector machine is used as the classifier of genetic algorithm feature selection to evaluate the subset of features related to the survival rate of patients. The main process of multi-index feature extraction based on genetic algorithm feature selection (GA-FS) is as follows.

**Figure 1:**
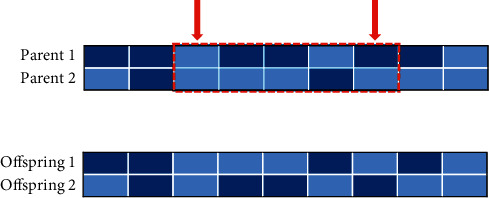
Principle of crossover operator.

Step 1: the generation of the initial population
A population is randomly generated as the first-generation solution of the problem. 17 blood indicators and TNM staging information of 360 esophageal cancer patients are selected as inputs and normalized to [−1,1] by the mapminmax function. The mapminmax function is calculated by the following equation:
  (1) $$\begin{array}{c} y=\frac{\left(y_{\max}-y_{\min}\right)\left(x-x_{\min}\right)}{\left(x_{\max}-x_{\min}\right)}+y_{\min}, \end{array}$$
   where *y*_max_ is 1 and *y*_min_ is −1.
Step 2: coding individuals in the population
The chromosome of each individual in the population is coded using a binary coding method, and each binary bit corresponds to each feature in the feature set. The initial characteristics include seventeen blood indicators, T staging, N staging, and TNM staging. In the value of each bit of the binary code, “0” indicates that the feature is not selected, and “1” indicates that the feature is selected. The dataset is divided into training set and test set.
Step 3: determine the fitness function
The value of the fitness function indicates the pros and cons of the individual or solution. The purpose of genetic algorithm (GA) used for feature selection is to improve the classification accuracy of the least-squares support vector machine (LSSVM) while reducing the number of selected features as much as possible. Therefore, the fitness function is constructed as Fitness=*α* · *R*+*β* · *M*/*N*. *R* is the classification accuracy of the LSSVM classifier. *M* is the number of selected features. *N* is the number of all features. *α* is a scaling parameter, which reflects the proportion of classification accuracy in the fitness function. *β* is the parameter importance, which reflects the weight of the selected number of features in the fitness function, and *α*+*β*=1.
Step 4: sort and select
The fitness values are calculated and individuals in the population are selected using a roulette wheel algorithm as a selection operator. The greater the fitness (i.e., the higher the classification accuracy and the lower the number of features), the greater the probability that the individual will be selected for the next generation.
Step 5: crossover
In this study, the crossover operation uses a two-point crossover operator, and the principle of the crossover operator is shown in Figure 1. Two crossover points are randomly set in the individual code string, and then, part of the gene exchange is performed. The crossover probability is generally 0.4 to 0.99, and the crossover probability selected in this study is 0.7.
Step 6: mutation
Under the condition of meeting the set mutation probability, the individuals in the population are sequentially subjected to random bit mutation. In the genetic algorithm (GA), the value of the mutation probability is generally 0.001 to 0.1, and the mutation probability used in this study is 0.05.
Step 7: the fitness value is calculated
The selected features are input into the LSSVM, and the fitness value is obtained by the ten-fold cross-validation method. If the current solution is better than the optimal solution, the optimal solution is updated.
Step 8: Step 3 is cycled to Step 7.

When the maximum number of iterations is reached, the loop ends. To clearly express the GA-FS process, the framework of GA-FS is shown in Algorithm 1.

Through the feature selection results of genetic algorithm, the index combinations that are more relevant to patient survival can be obtained: T staging, N staging, TNM staging, WBC, EO, RBC, PLT, TP, PT, and INR. At this time, the ten-fold cross-validation classification accuracy of LSSVM reaches the highest, and the value is 83.077 %.

### 2.3. The Correlation of Indicators Is Verified by Cox Regression Analysis

**Figure 2:**
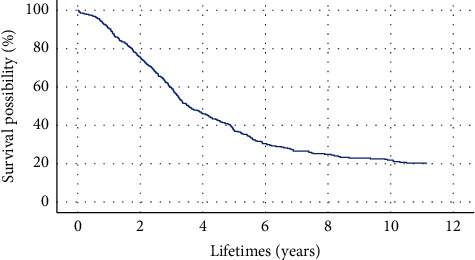
Survival function at the mean of the covariate. The survival years are taken as the time, and the ten indicators obtained from genetic algorithm feature selection are used as covariates.

The Cox regression model is a semiparametric regression model that can analyze the impact of multiple factors on survival [34]. Therefore, it is widely used in the medical field. The “SPSS 22.0” statistical software is used to make the Cox model. The survival time and survival outcome of patients with ESCC are used as dependent variables. The above ten indicators are independent variables. The survival function at the mean of the covariate is shown in Figure 2. The results show that the *p* value of the overall score of the ten indicators is 0.000131 far less than 0.05. The combination of these ten indicators is significantly related to the survival rate of patients.

### 2.4. Evaluation and Establishment of Prognostic Indicators

This section establishes and evaluates the prognostic index (PI) of patients with ESCC to better classify patients and provide good clinical guidance. In the above section, the ten indicators that are significantly related to the survival of patients are selected through genetic algorithm feature selection, which are T stage, N stage, TNM stage, WBC, EO, RBC, PLT, TP, PT, and INR. The binary logistic regression analysis [35] is used to construct the prognostic index. The patient's survival status is used as the dependent variable, and ten indicators are used as independent variables. The prognostic index of ESCC is constructed by the following equation:

(2) $$\begin{array}{c} PI=0.481*\text{TNM}-0.809*\text{INR}. \end{array}$$

**Figure 3:**
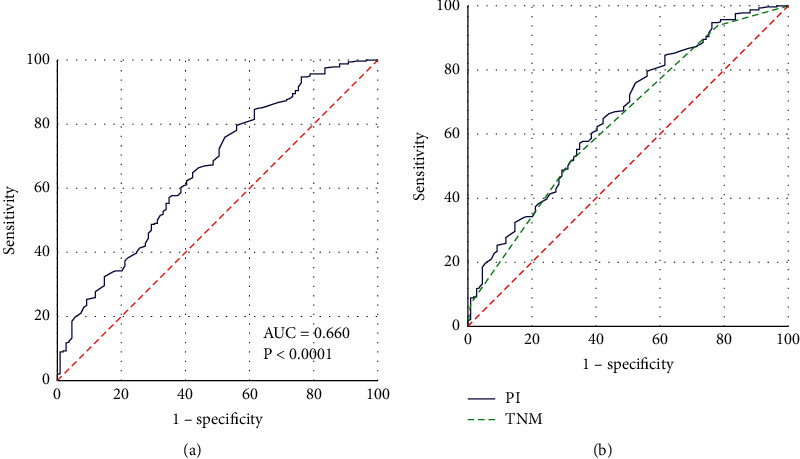
ROC analysis of PI and TNM. (a) ROC analysis of PI. (b) Comparative analysis of ROC for PI and TNM. The horizontal coordinate is “1-specificity,” and the vertical coordinate is “sensitivity.” The larger the area under the curve, the stronger the significance.

The receiver operating characteristic (ROC) [36] curve is usually used to select the best diagnostic threshold and divide the indicators into two categories. The ROC curve of PI is shown in Figure 3(a). The AUC value is 0.660, *p* < 0.001, indicating that PI has a high predictive value for the prognosis of ESCC patients. The comparison of ROC curves between PI and TNM staging systems is shown in Figure 3(b). The comparison results of PI and TNM are shown in Table 3. By analyzing and comparing the ROC curves of PI and TNM, it can be concluded that the predictive effect of the prognostic index PI in this study is better than that of the TNM staging system.

To better predict the survival status of ESCC patients, the ROC curve is further analyzed to determine the best cutoff value of PI. The PI values of all samples are used as inputs, and the ROC curve is drawn, as shown in Figure 3. The value of the area under the curve is 0.660, which is greater than 0.5, *P* < 0.001. Obviously, there is a threshold for PI. By calculating the Youden index, PI can be divided into two levels. The Youden index is calculated by the following equation:

(3) $$\begin{array}{c} \text{Youden index}=\text{Sensitivity}-\left(1-\text{Specificity}\right). \end{array}$$

**Figure 4:**
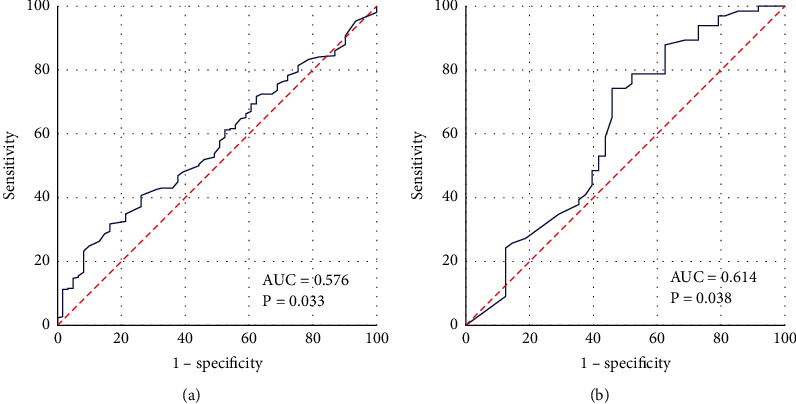
ROC analysis for dividing PI staging. (a) ROC for high PI samples. (b) ROC for low PI samples. The horizontal coordinate is “1-specificity,” and the vertical coordinate is “sensitivity.” The larger the area under the curve, the stronger the significance.

The Youden index is calculated as 0.303. The Youden index, AUC value, significance, and other related indicators are shown in Table 4. Then, for samples with PI values higher than 0.303 and samples with PI values lower than 0.303, ROC curves are drawn, as shown in Figure 4. The Youden index, AUC value, significance, and other related indicators are shown in Table 4. It can be seen from Table 4 that the AUC values of the three ROC curves are all greater than 0.5, and the significance *P* value is less than 0.05.

**Figure 5:**
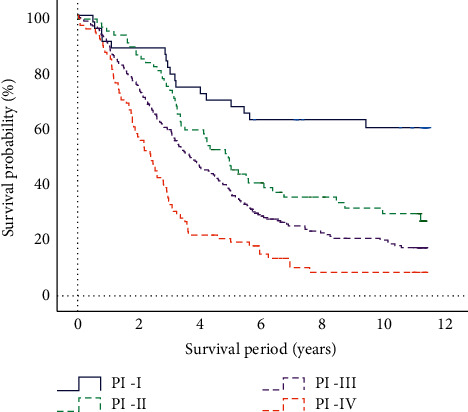
Kaplan–Meier survival analysis of PI stages.

According to the ROC curve, the three critical thresholds of PI can be obtained in sequence. The three critical thresholds are 0.303, 0.016, and 0.873, respectively. According to the critical threshold, PI is divided into four stages, namely PI-I, PI-II, PI-III, and PI-IV. The four stages of PI are analyzed by the Kaplan–Meier, and the results are shown in Figure 5. According to the Kaplan–Meier analysis [37], PI-I has the best prognostic effect, which is better than PI-II, PI-III, and PI-IV for patients with ESCC.

### 2.5. Divide Risk Levels Based on Patient's Age

**Figure 6:**
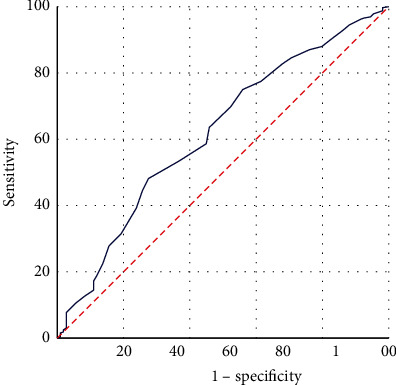
ROC analysis of age.

At present, age is considered by most studies to be an important factor affecting the prognosis of ESCC. The age factor has an important influence on the physiological immunity of the patient, and it is related to the patient's tolerance to different treatment methods. Therefore, differences in age factors will also lead to different prognoses of ESCC patients. It is important to construct different survival prediction models for patients of different ages. The ROC curve is used to determine the best cutoff value of the patient's age. It is plotted with the age of all samples as the variable, named “ROC of the patient's age,” as shown in Figure 6. The area under the curve (AUC) value is 0.618, which is greater than 0.5, and *P* < 0.001. Obviously, a critical threshold can be found for age, which divides age into two risk levels.

**Figure 7:**
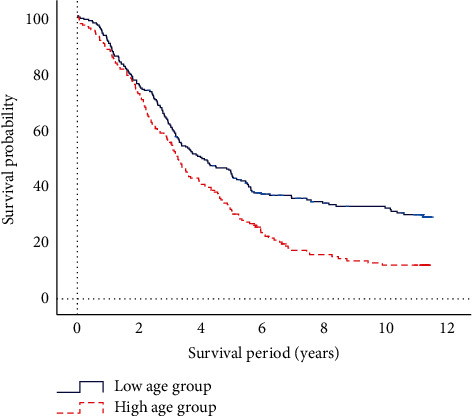
Kaplan–Meier survival analysis of age.

After calculating the Youden index, the critical threshold of age is 61.5 years. By calculating critical thresholds, patients are divided into the high- and low-age groups. The Kaplan–Meier survival analysis is performed based on the high- and low-value groups of age, and the results are shown in Figure 7. There is a significant difference between the high-age group and the low-age group (*P* < 0.05) on survival rate, and the low-age group has a better survival rate than the high-age group.

## 3. Survival Prediction Based on LSSVM

### 3.1. Bald Eagle Search Algorithm-Least-Squares Support Vector Machine

The bald eagle search algorithm (BES) is proposed by Alsattar et al. [38]. It is a meta-heuristic optimization algorithm based on the behavior strategy or social behavior of the bald eagle during hunting. The algorithm has strong global search capabilities and can effectively solve various complex numerical optimization problems. In this study, the bald eagle search algorithm is used to optimize the parameters of the least-squares support vector machine, which improved the prediction accuracy of the least-squares support vector machine. The survival rate of ESCC patients is predicted based on the proposed BES-LSSVM classification prediction model.

The bald eagle search algorithm is mainly divided into three stages, namely select stage, search stage, and swooping stage.

#### 3.1.1. Select Stage

In the select stage, the bald eagles will select the best area (according to the amount of food) within the selected search area and start looking for prey. At this time, the position *P* of the bald eagle is determined by multiplying the a priori information of the random search by *α*. The mathematical model of this behavior is constructed as follows:

(4) $$\begin{array}{c} P_{i,\text{new}}=P_{\text{best}}+\alpha *r\left(P_{\text{mean}}-P_{i}\right). \end{array}$$

where *α* is used to control the position change parameter within the range of (1.5, 2); *r* is a random number between (0,1). *P*_best_ represents the best position of the bald eagle based on the previous search. *P*_mean_ is the average position of the bald eagle after the previous search. *P*_*i*_ represents the position of the *i*th bald eagle.

#### 3.1.2. Search Stage

In the search stage, the bald eagles fly in different directions in a spiral shape, speeding up the search for prey. Then, the bald eagle will look for the best position in the selected space to swoop and hunt. The position update of the bald eagle during spiral flight adopts the form of polar coordinate equation, as follows:

(5) $$\begin{array}{c} \left\{\begin{array}{c} x\left(i\right)=\frac{xr\left(i\right)}{\max\left(\left|xr\right|\right)},\\ y\left(i\right)=\frac{yr\left(i\right)}{\max\left(\left|yr\right|\right)},\\ xr\left(i\right)=r\left(i\right)*\;\sin\left(\theta \left(i\right)\right),\\ yr\left(i\right)=r\left(i\right)*\;\cos\left(\theta \left(i\right)\right),\\ \theta \left(i\right)=\alpha *\pi *\text{rand,}\\ r\left(i\right)=\theta \left(i\right)+R*\text{rand,} \end{array}\right) \end{array}$$

where *a* and *R* are the parameters in the range of (5,10) and (0.5, 2), respectively, which are used to control the spiral regression trajectory. *θ*(*i*) and *r*(*i*) are the polar angle and polar diameter of the spiral equation, respectively. *x*(*i*) and *y*(*i*) represent the position of the bald eagle in polar coordinates, and the values are both (−1,1). *xr*(*i*) and *yr*(*i*) represent the position of the bald eagle in the Cartesian coordinate system. rand is a random number (0,1).

The location of the bald eagle is constructed as follows:

(6) $$\begin{array}{c} P_{i,\text{new}}=P_{i}+y\left(i\right)*\left(P_{i}-P_{i+1}\right)+x\left(i\right)*\left(P_{i}-P_{\text{mean}}\right). \end{array}$$

#### 3.1.3. Swooping Stage

In the swooping stage, the bald eagles quickly swoop from the best position in the search space to their target prey. At the same time, other individuals in the population move to the best position and attack the prey. The state of motion of the bald eagle is described by the polar coordinate equation:

(7) $$\begin{array}{c} \left\{\begin{array}{c} \theta \left(i\right)=\alpha *\pi *\text{rand,}\\ r\left(i\right)=\theta \left(i\right),\\ xr\left(i\right)=r\left(i\right)*\text{sinh}\left(\theta \left(i\right)\right),\\ yr\left(i\right)=r\left(i\right)*\text{coxh}\left(\theta \left(i\right)\right),\\ x1\left(i\right)=\frac{xr\left(i\right)}{\max\left(\left|xr\right|\right)},\\ y1\left(i\right)=\frac{yr\left(i\right)}{\max\left(\left|yr\right|\right)}. \end{array}\right) \end{array}$$

The formula for updating the position of the bald eagle during swooping is constructed as follows:

(8) $$\begin{array}{c} \left\{\begin{array}{c} \delta_{x}=x1*\left(P_{i}-c_{1}*P_{\text{mean}}\right),\\ \delta_{y}=y1*\left(P_{i}-c_{2}*P_{\text{best}}\right),\\ P_{i\text{,new}}=\text{rand}*P_{\text{best}}+\delta_{x}+\delta_{y}, \end{array}\right) \end{array}$$

where *c*_1_ and *c*_2_ increase the exercise intensity of the bald eagle to the optimal point and the center point, and the value range is (1,2).

For LSSVM, the choice of kernel function is a key factor. The RBF kernel function is selected in this study, and the *RBF* kernel function can be expressed as follows:

(9) $$\begin{array}{c} K\left(x,z\right)=\exp\left(-g{\left\|x-z\right\|}^{2}\right),g>0, \end{array}$$

where *g* is the parameter coefficient of the kernel function, which affects the performance of LSSVM.

In this study, to improve the classification accuracy of LSSVM, BES is selected to optimize the penalty factor *c* and the kernel function parameter *g* of LSSVM. The classification error rate of LSSVM is used as the objective function of BES optimization, and the objective function is fitness function  = 1 − classification error rate. The larger the fitness value, the higher the classification effect of LSSVM.

To clearly express the BES-LSSVM process, the framework of BES-LSSVM is shown in Algorithm 2.

### 3.2. Survival Prediction of Esophageal Squamous Cell Carcinoma

**Figure 8:**
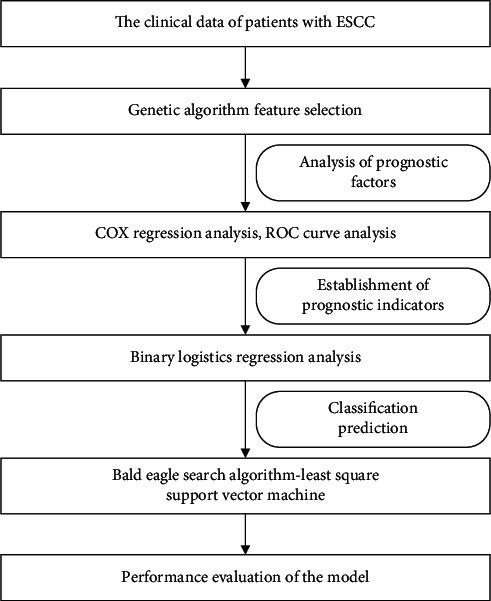
Framework of the overall implementation of the survival prediction model for patients with ESCC.

Ten indicators related to the survival rate of ESCC patients are obtained through the method of genetic algorithm feature selection. These indicators are T stage, N stage, TNM stage, WBC, EO, RBC, PLT, TP, PT, and INR. The prognostic index PI of ESCC patients is obtained by the binary logistic regression. The eleven indicators of patients are used as inputs to the BES-LSSVM model, and the five-year survival rate of the patients is used as the output. Survival prediction models for ESCC patients in the high-age group and the low-age group are established separately. The framework of the overall implementation of the survival prediction model for patients with ESCC is shown in Figure 8. To verify the validity of this model, grasshopper optimization algorithm-least-squares support vector machine (GOA-LSSVM) [39], particle swarm optimization-least-squares support vector machine (PSO-LSSVM) [40], differential evolution-least-squares support vector machine (DE-LSSVM) [41], sparrow search algorithm-least-squares support vector machine (SSA-LSSVM) [42], bald eagle search-back propagation neural network(BES-BPNN), and bald eagle search-extreme learning machine(BES-ELM) are used for comparison.

For the parameter setting of the bald eagle search algorithm, the bald eagle population number is set to 20, and the number of iterations is set to 100. For the particle swarm algorithm, both *c*_1_ and *c*_2_ are set to 1.5. The population size is set to 20, and the number of iterations is set to 100. For the grasshopper optimization algorithm, the population size is set to 20, and the maximum number of iterations is set to 100. For differential evolution algorithm, the scaling factor *F* is set to 0.5, the crossover probability CR is set to 0.9, and the maximum number of iterations is set to 100. For the sparrow search algorithm, the population size is set to 20, the safety value is set to 0.6, and maximum number of iterations is set to 100. The dataset is divided into ten parts, and the ten-fold cross-validation method is used to verify the performance of the model. Nine samples are used as the training set, and one sample is used as the validation set. The cross-validation is repeated 10 times, and the average of the ten results is obtained. This method enables training and testing with random samples repeatedly, and the results are verified once each time. The effect of boundary patient data on the performance of the least-squares support vector machine is effectively reduced. The evaluation metrics include classification accuracy, sensitivity, specificity, and running time. Among them, sensitivity is a measure of the model's ability to identify positive samples and specificity is a measure of the model's ability to identify negative samples. Sensitivity and specificity are calculated as follows:

(10) $$\begin{array}{c} \left\{\begin{array}{c} \text{Sensitivity}=\frac{\text{TP}}{\text{TP}+\text{FN}}\text{,}\\ \text{Specificity}=\frac{\text{TN}}{\text{TN}+\text{FP}}, \end{array}\right) \end{array}$$

where true positive (TP) is the number of positive samples correctly identified, true negative (TN) is the number of negative samples correctly identified, false positive (FP) is the number of positive samples incorrectly identified, and false negative (FN) is the number of positive samples incorrectly identified. The prediction results of the LSSVM optimized by the five optimization algorithms, BES-BPNN, and BES-ELM model are shown in Table 5. The optimal LSSVM model parameters under different optimization methods are shown in Table 6.

It can be seen from Table 5 that in the high-age group, the prediction accuracy of BES-LSSVM, GOA-LSSVM, DE-LSSVM, PSO-LSSVM, SSA-LSSVM, BES-BPNN, and BES-ELM is 86.538%, 85.769%, 85.384%, 84.615%, 86.154%, 83.902%, and 83.477%, respectively. In the low-age group, the prediction accuracy of BES-LSSVM, GOA-LSSVM, DE-LSSVM, PSO-LSSVM, SSA-LSSVM, BES-BPNN, and BES-ELM is 86.495%, 85.435%, 85.217%, 84.782%, 85.843%, 83.479%, and 83.913%, respectively. The comparison shows that BES-LSSVM has a high accuracy rate and can accurately predict the five-year survival rate of ESCC patients. In terms of sensitivity and specificity, the proposed BES-LSSVM also outperforms other models. Besides, it can be seen from Table 5 that BES-LSSVM has the fastest running time.

To better demonstrate the effectiveness of the proposed model, the Wisconsin Diagnostic Breast Cancer (WBCD) dataset is used for testing, and the results are shown in Table 7. From the test results, it can be seen that BES-LSSVM has higher prediction accuracy and faster running time than other models. Therefore, the survival status of cancer patients can be effectively predicted by the survival prediction model proposed in this study.

## 4. Conclusions

To accurately and effectively predict the five-year survival rate of patients with ESCC, a survival prediction model based on genetic algorithm feature selection, binary logistic regression, and least-squares support vector machine is proposed in this study. A genetic algorithm and Cox regression are used to determine ten indicators that are significantly related to the survival of patients with ESCC. Based on the binary logistic regression, a prognostic indicator PI with predictive value is constructed. Patients are divided into the high-age groups and the low-age groups by ROC curve analysis. Through the Kaplan–Meier survival analysis, it is concluded that the low-age group has a better survival rate than the high-age group. The bald eagle search algorithm-least-squares support vector machine (BES-LSSVM) is further proposed, which effectively predicts the five-year survival rate of patients with ESCC. The accuracy of BES-LSSVM in predicting the five-year survival of patients with ESCC is better than the existing GOA-LSSVM, PSO-LSSVM, DE-LSSVM, SSA-LSSVM, BES-BPNN, and BES-ELM. This reflects the good practical value of the ESCC survival prediction model proposed in this study in the field of cancer classification prediction.

However, the accuracy of the model may be affected by increase in number of samples and classes. Moreover, sometimes, it is a possibility that during the feature selection process, few important features are discarded. In the future, the combination of swarm intelligence optimization algorithm and the latest deep learning models (such as deep neural network and convolutional neural network) will be used to develop a new survival prediction model for patients with ESCC on a larger and more complex dataset.

## Figures and Tables

**Algorithm 1:**
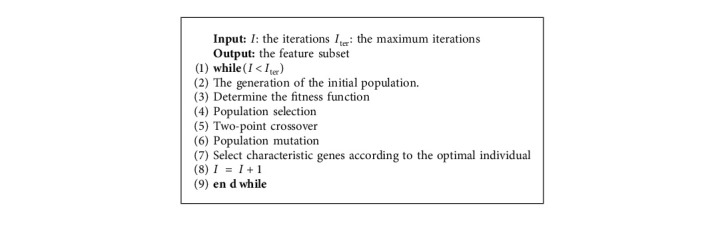
Framework of GA-FS.

**Algorithm 2:**
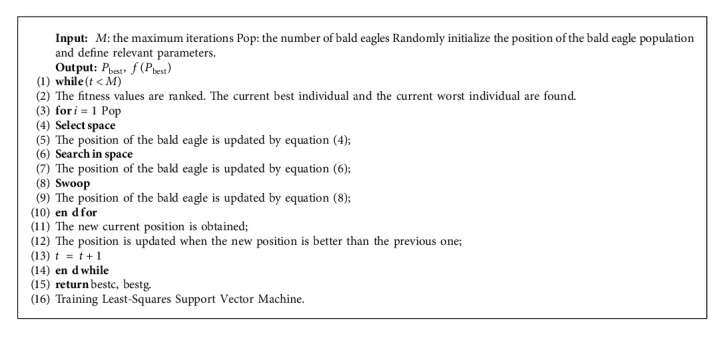
Framework of BES.

**Table 1:** Population proportion information of the dataset.

Project	Category	Number of population	Percentage of population (%)
Genders	Male	222	61.7
	Female	138	38.3

Ages	≤61.5	230	63.9
	>61.5	130	36.1

T stages	T1	54	15
	T2	99	27.5
	T3	205	56.9
	T4	2	0.1

N stages	N0	191	53.1
	N1	103	28.6
	N2	48	13.3
	N3	18	5

TNM stages	I	47	13.1
	II	156	43.3
	III	137	38.1
	IV	20	5.6

**Table 2:** Basic information about seventeen blood indicators.

Variable	Mean	Median (range)	Variance	Standard deviation
WBC	6.633	6.2 (2.5–13.6)	4.427	2.104
LYMPH	1.869	1.9 (0–4)	0.401	0.633
GLOB	29.306	29 (17–45)	27.160	5.212
PT	10.327	10.3 (7–16.6)	2.690	1.640
ALB	42.011	42 (24–56)	27.259	5.212
RBC	4.430	4.45 (2.6–6.04)	0.234	0.483
TT	15.304	15.5 (1.3–21.3)	3.583	1.893
BASO	0.042	0 (0–1)	0.007	0.082
EO	0.137	0.1 (0–3)	0.044	0.209
INR	0.795	0.79 (0.45–1.64)	0.033	0.181
NEUT	4.033	3.7 (0.3–17)	3.491	1.868
TP	71.428	71 (50–92)	53.064	7.285
MONO	0.405	0.4 (0–1.3)	0.069	0.263
FIB	379.431	367.85 (189.5–774.43)	924.038	30.398
HGB	138.311	139 (63–189)	218.705	14.789
PLT	239.781	232.5 (51–576)	52.606	7.253
APTT	36.112	35.25 (15.4–78.5)	60.110	7.753

The unit of WBC, LYMPH, GLOB, ALB, RBC, BASO, EO, NEUT, TP, HGB, and PLT is g/L. The unit of PT, TT, and APTT is second(s). The unit of FIB is mg/L.

**Table 3:** Results of ROC analysis for PI and TNM.

Project	Sensitivity	Specificity	AUC	Significance level *P*
PI	0.796	0.440	**0.660**	<**0.0001**
TNM	0.515	0.679	0.639	<0.0001

**Table 4:** Results of ROC curve analysis for PI critical threshold.

Project	ROC for all PI samples	ROC for low PI samples	ROC for high PI samples
Area under the ROC curve (AUC)	**0.660**	**0.614**	**0.576**
Standard error	0.030	0.056	0.033
95% confidence interval	0.600 to 0.719	0.505 to 0.723	0.511 to 0.642
Significance level *P*	<**0.0001**	**0.038**	**0.029**
Youden index	0.237	0.284	0.158
Associated criterion	**0.303**	**0.016**	**0.873**
Sensitivity	0.796	0.742	0.309
Specificity	0.440	0.542	0.848

**Table 5:** Comparison of different algorithms for predicting five-year survival of patients with esophageal squamous cell carcinoma.

	Algorithm	10-fold cross-validation accuracy (%)	Sensitivity (%)	Specificity (%)	Running time (s)
High-age group	BES-LSSVM	86.538	88.032	86.437	1.661
	GOA-LSSVM	85.769	86.971	85.101	3.464
	DE-LSSVM	85.384	86.626	84.668	8.123
	PSO-LSSVM	84.615	85.397	83.537	3.641
	SSA-LSSVM	86.154	87.329	85.553	2.875
	BES-BPNN	83.902	85.673	83.393	10.615
	BES-ELM	83.477	84.419	82.907	6.171

Low-age group	BES-LSSVM	86.495	88.327	85.991	1.846
	GOA-LSSVM	85.435	87.229	84.915	4.254
	DE-LSSVM	85.217	86.802	84.474	9.950
	PSO-LSSVM	84.782	86.595	84.245	3.846
	SSA-LSSVM	85.843	87.675	85.338	3.412
	BES-BPNN	83.479	85.271	82.959	11.743
	BES-ELM	83.913	85.706	83.393	7.036

**Table 6:** Optimal LSSVM model parameters under different optimization algorithms.

Algorithm	High-age group		Low-age group	
	Penalty factor	Kernel function parameter	Penalty factor	Kernel function parameter
BES-LSSVM	77.946	2.090	60.290	2.493
GOA-LSSVM	54.429	1.225	22.895	0.106
DE-LSSVM	66.155	1.044	50.816	0.735
PSO-LSSVM	61.902	1.086	46.111	0.459
SSA-LSSVM	77.217	10.192	75.204	5.991

**Table 7:** Comparison of the results of different algorithms.

Algorithm	10-fold cross-validation accuracy (%)	Sensitivity (%)	Specificity (%)	Running time (s)
BES-LSSVM	97.01	98.19	95.09	2.793
GOA-LSSVM	96.28	97.75	93.90	7.263
DE-LSSVM	96.10	97.64	93.62	6.824
PSO-LSSVM	96.27	97.75	93.89	5.772
SSA-LSSVM	96.65	97.97	94.54	3.818
BES-BP	95.26	97.11	92.34	12.749
BES-ELM	95.61	97.33	92.88	7.837

## Data Availability

The data used to support the findings of the study can be obtained from the corresponding author upon request.
